# Supplementary material for: InSillyClo, a User-Friendly Web Application to Assist Large-Scale Golden Gate Cloning and MoClo Workflows
Source: ACS Synth Biol. 2025 Dec 17;15(1):353–8. doi: 10.1021/acssynbio.5c00553 (PMC12814555; doi:10.1021/acssynbio.5c00553)
Supplement: Supplementary file 1 [file sb5c00553_si_001.pdf]

InSillyClo, a user-friendly web application to assist large-scale Golden Gate Cloning and MoClo workflows

Henri Galez, Bryan Brancotte, Juliette Bonche, Julien Fumey, Sara Napolitano, Gregory Batt*

* To whom correspondence may be addressed. Email: gregory.batt@inria.fr

Summary

Supplementary Text S1: Definitions and explanations of terms

Supplementary Text S2: Guidelines for quick cloning with InSillyClo

Supplementary Text S3: Guidelines for generation of InSillyClo input files

Supplementary Text S4: Description of the campaign data produced by InSillyClo

Supplementary Text S5: Standard typed cloning workflow

Supplementary Text S6: InSillyClo with other MoClo systems

Supplementary Text S7: Local implementation with the command line tool

Text S1: Definitions and explanations of terms

Cloning workflow: Set of dry and wet-lab tasks from the decision to construct a set of plasmids to the verification of these plasmids once assembled.

Cloning campaign: Instantiation of a cloning workflow with a large number of plasmids requiring significant time for preparation and realization. Typically, users would build genetic circuits with combinatorial variations of some genetic elements resulting in a set of Golden Gate reactions with both shared and unshared input parts.

Input plasmids (iPs): Plasmids mixed together in the Golden Gate reaction mix. They are digested by the restriction enzyme and ligated together to form the output plasmid.

Output plasmid (oP): Plasmid obtained in a Golden Gate reaction from the assembly of the input plasmids.

Input part: Input parts have a biological meaning (e.g. *Promoter*) while input plasmids only refer to the DNA sequence. InSillyClo uses the notion of type to refer to this biological meaning. In raw assemblies, users assemble together input plasmids irrespectively of what they contain. In typed assembly, users assemble input parts creating a meaningful biological sentence (e.g. *Promoter*, *Gene* and *Terminator*). This requires assigning a type to each input plasmids so that they belong to the appropriate input part.

Assembly type: Golden Gate Cloning technology refers to the assembly of input plasmids, and can be seen as a raw assembly. Modular Cloning adds biological meaning to the assembly by adding a grammar, defined by the input parts it contains. The biological meaning of an assembly, or its particular grammar, is rather specific to each MoClo kit. Therefore, it is important to be able to define a tailored assembly for each MoClo grammar, which is done by generating new assembly types.

Assembly template: File containing all the information of an assembly type. Users fill it in with genetic designs to be constructed.

Output separator: Character (e.g. comma ",") separating the input part name during the computation of the output name.

Input separator: Character (e.g. comma ",") separating input subparts within an input part. For instance, using the comma as separator, the script will understand that in *GFP,FLAG* it should look for the input part *GFP* and *FLAG* separately (i.e. encoded in two different plasmids).

Text S2: Guidelines for quick cloning with InSillyClo

For users primarily interested in the automation of Golden Gate reactions, a minimal version of the workflow is possible:

1. Download the raw assembly template https://insillyclo.pasteur.cloud/assembly/8/
    - If more than 8 input plasmids, additional columns can be added directly in the template
    - Edit the restriction enzyme if other than BsaI
2. Prepare a *SequenceDB.zi*p archive with plasmids in Genbank format (only .gb files)
3. Fill in the template with genetic designs using the Genbank file names of the input plasmids
    - Since specification is made using file names, *iP_mapping* file can be omitted
4. Upload on Assembly Simulator module the filled template and the zip archive
5. Run the simulation and generate the campaign data

Text S3: Guidelines for generation of InSillyClo input files

First, new users need to define their assembly types and naming conventions. Doing so collectively at the laboratory level during the InSillyClo initialization phase ensures consistency and good connections with the existing information management system. This process can also help identify existing inconsistencies.

New users find below help for generating InSillyClo files, including the database of input plasmids, the iP mapping file, the assembly template file, and the database of primers for PCR as verification (optional).

Database of input plasmid sequences – zip archive of Genbank files

Users need to gather the input plasmid sequences in a folder as Genbank files (.gbk not supported). For the command line tool, users provide the folder path, while for the web application, users must create a zip archive.

iP mapping file - csv format

This file links the identifier (pID) of each plasmid to its name and type. The algorithm uses the pID to match a plasmid name with its sequence; therefore, the pID and the Genbank filename must match. The pID-name combination, or the pID-name/type combination in typed assembly, should be unique and names should be as descriptive as possible. Of note, if two plasmids with different identifiers have the same name, the program will use the plasmid whose pID comes first in alphanumeric order. Using types is efficient to embed information in it rather than extending plasmid name (e.g. GFP of type 3a, rather than NterGFP). If using typed assembly, users need to define the typing system associated to their Moclo to be able to assign types to their input plasmids. They can take inspiration from the MoClo systems presented in Text S6.

Users can add an optional column, either “Mass Concentration” or “Mol Concentration” to specify the concentrations of input plasmids. The units are ng/µL or fmol/µL. Concentrations can also be added in the “Dilution” section of the “Result” page on the web application.

This file can be omitted if users enter directly Genbank filenames of the input plasmids in the Campaign file. Any spreadsheet program can be used to edit csv files.

Template file - xlsx format

This file contains all the information regarding the assembly, that is the name, the restriction enzyme and, the characteristics of all input parts. It must follow this structure:

	A	B	C	D	E	F	G	H
1	**Assembly settings**							
2	**Restriction enzyme**	BsaI						
3	**Name**	YTK_L1						
4	**Output separator**	-						
5								
6								
7								
8								
9	**Assembly composition**	**Part name ->**	ConL	Promoter	Gene	Terminator	ConR	Backbone
10		**Part types ->**	1	2,[2a, 2b]	3,[3a, 3b]	4,[4a, 4b]	5	678,[6, 7, 8]
11		**Is optional part ->**	True	False	False	False	True	False
12		**Part name should be in output name ->**	False	True	True	True	False	False
13		**Part separator ->**		.	.	.		.
14	**Output plasmid id ↓**	**OutputType (optional) ↓**	↓	↓	↓	↓	↓	↓
15								
16								
17								
18								

Figure 1: Template file of the Yeast Tool Kit Level 1 assembly.

This file can be generated in 2 different ways:

1. Running the Assembly Designer module of the web application (Text S5). Users fill in forms and generate the template which can then be downloaded. Of note, default types are assigned using number/number-letter (e.g. 1, 1a, 1b, 2…). Users need to open the file with a spreadsheet program to specify their types. They must match types used in the iP mapping file.
2. Running the *template* command of the command line tool (Text S7).

We advise users who want to mostly edit their assembly in a spreadsheet program to download the raw assembly template from the web application (https://insillyclo.pasteur.cloud/assembly/) and modify it in a spreadsheet program.

We used Excel and Google Sheet to edit the template file. In theory, any program able to open and save xlsx files can be used. OpenOffice Calc is not suitable because it cannot save xlsx file.

For each cloning campaign, users open this template file and fill it with plasmids to be assembled, thereby creating the Campaign file. We recommend renaming the file with the name of the cloning campaign before saving it to keep the template clean for the next campaign.

DB primer file – csv format (needed only for verification by PCR)

A csv file with two columns entitled "primerID" and "sequence". The primer ID is entered in the verification section of the Assembly Simulator result page. When giving a primer pair in this section, for instance "P29,P30", make sure not to have a space character after the comma.

Text S4: Description of the campaign data produced by InSillyClo

Here, we present the output data of the InSillyClo workflow with a untyped/raw assembly of the fluorescent proteins Venus with several promoters.

DB_produced_plasmid.csv

Simple file containing automatically generated names of the output plasmids and their types.

	A	B	C
1	pID	Name	Type
2	pSA001	AmpRS1-pTDH3-Venus-tADH1	
3	pSA002	AmpRS1-pTEF2-Venus-tADH1	
4	pSA003	AmpRS1-pALD6-Venus-tADH1	
5	pSA004	AmpRS1-pREV1-Venus-tADH1	

Figure 2: Example pf DB_produced_plasmid.csv for a raw assembly.

Output plasmid sequences

InSillyClo automatically computes the assembly of the input sequences into output sequences which are then given in Genbank format. Of note, annotations present in the input files are kept during the assembly.

Name	Type
pSA001	GenBank DNA
pSA002	GenBank DNA
pSA003	GenBank DNA
pSA004	GenBank DNA

Figure 3: Genbank files containing the assembled DNA sequences.

slowpoke-combination-to-make.csv (or auto-gg-combination-to-make.csv in past versions)

This file is used by Slowpoke tool to generate the protocol containing the instructions given to Opentrons robot OT-2 or Flex to perform the Golden Gate Cloning. The additional files needed by the tool are csv tables indicating the localization of input plasmids in a 96 well-plate format and the python protocol.

	A	B	C	D	E	F
1	pSA001	AmpRS1-pTDH3-Venus-tADH1	pMYT039	pYTK009	pYTK033	pYTK053
2	pSA002	AmpRS1-pTEF2-Venus-tADH1	pMYT039	pYTK014	pYTK033	pYTK053
3	pSA003	AmpRS1-pALD6-Venus-tADH1	pMYT039	pYTK018	pYTK033	pYTK053
4	pSA004	AmpRS1-pREV1-Venus-tADH1	pMYT039	pYTK027	pYTK033	pYTK053

Figure 4: Example of slowpoke-combination-to-make.csv

Dilutions

InSillyClo computes dilutions of input plasmids needed to mix them in equimolar quantity. Users need to specify input plasmid concentrations and cloning reaction parameters. Besides key parameters of the reaction (i.e. final reaction mix volume, molar quantity of input plasmids, and volume of enzyme/buffer), it is also possible to choose parameters to facilitate pipetting like the minimal volume accepted to be pipetted, or the minimal remaining volume in the master-mix. Additionally, users can choose between several dilution protocols generated:

- Direct dilution: take directly the volume needed of the input plasmid to add to the reaction mix. Of note, the buffer column represents volume for both the enzymes and the buffer.

	A	B	C	D	E	F	G	H	I	J
1	plasmid_id	h2o_volume	buffer	pMYT039	pYTK009	pYTK014	pYTK018	pYTK027	pYTK033	pYTK053
2	pSA001	7.704	2	0.119	0.063				0.082	0.032
3	pSA002	7.524	2	0.119		0.244			0.082	0.032
4	pSA003	7.7	2	0.119			0.068		0.082	0.032
5	pSA004	7.7	2	0.119				0.067	0.082	0.032

Figure 5: Example of direct dilution.

We also automated the calculation of a master-mix, which is made of water, enzymes, buffer, and the plasmids present in every assembly (i.e. pMYT039, pYTK033 and pYTK053 in the example).

	A	B	C	D	E	F	G	H	I	J	K
1	plasmid_id	h2o_volume	buffer	mastermix	pMYT039	pYTK009	pYTK014	pYTK018	pYTK027	pYTK033	pYTK053
2	mastermix	0	8		0.476					0.327	0.127
3	pSA001	7.704		2.232		0.063					
4	pSA002	7.524		2.232			0.244				
5	pSA003	7.7		2.232				0.068			
6	pSA004	7.7		2.232					0.067		

Figure 6: Example of direct dilution with master-mix.

- 10x dilution: an intermediate dilution is made for each plasmid to avoid pipetting small volumes. We recommend setting parameters so the intermediate dilution is 10x compared to the final mix. The table generated is pretty large but can be easily reformatted in a spreadsheet program.

First, the intermediate dilutions and the master-mix are presented. In the example, parameters were chosen so that intermediate dilutions are made by adding 1µL of plasmid stock and then the volume of water needed to reach a 10x concentration. For instance, 1µL of pYTK009 is mixed with 7.4µL of water to obtain pYTK009_10x. Then, since the final volume is 10µL, we use 1µL of pYTK009_10x in the pSA001 assembly.

	A	B	C	D	E	F	G	H	I	J	K	L	M
1	plasmid_id	h2o_volum	buffer	pMYT039__	pYTK033__10x__	pYTK053__10x__	pMYT039	pYTK009	pYTK014	pYTK018	pYTK027	pYTK033	pYTK053
2	mastermix	0	8	4	4	4							
3	pMYT039__10x__	7.407					1						
4	pYTK009__10x__	14.765						1					
5	pYTK014__10x__	3.096							1				
6	pYTK018__10x__	13.676								1			
7	pYTK027__10x__	13.882									1		
8	pYTK033__10x__	11.246										1	
9	pYTK053__10x__	30.518											1

Figure 7: Example of 10x/intermediate dilution with master-mix (1/2).

Then comes the final reactions.

11	plasmid_id	h2o_volume	buffer	mastermix	pYTK009__10x__	pYTK014__10x__	pYTK018__10x__	pYTK027__10x__
12	pSA001	4		5	1			
13	pSA002	4		5		1		
14	pSA003	4		5			1	
15	pSA004	4		5				1

Figure 8: Example of 10x/intermediate dilution with master-mix (2/2).

Verification by PCR or restriction-digestion

The colony PCR simulation produces a gel with all the output plasmids. Correct bands appear in black (i.e. 2261 and 2281 bands). The algorithm tries to make an amplification on the input plasmids, which could notably happen on the backbone if the chosen primers bind on each side of the drop-out cassette. These bands representing bad clones appear light gray on the gel. The tool also simulates restriction digestion.

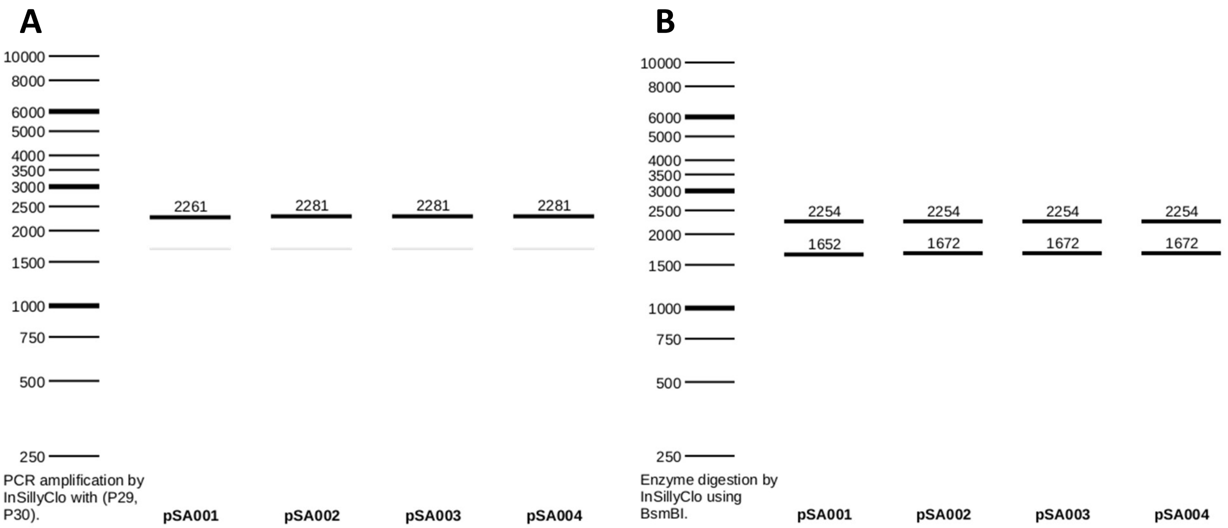

Figure 9: Agarose gel simulation for (A) colony PCR and (B) restriction digestion.

SBOL

InSillyClo computes a SBOL file in xml format tracking the assembly.

Text S5: Standard typed cloning workflow

Initialization

1. Define the assembly on Assembly Designer or directly use a public assembly (https://insillyclo.pasteur.cloud/assembly/), and download the associated template file

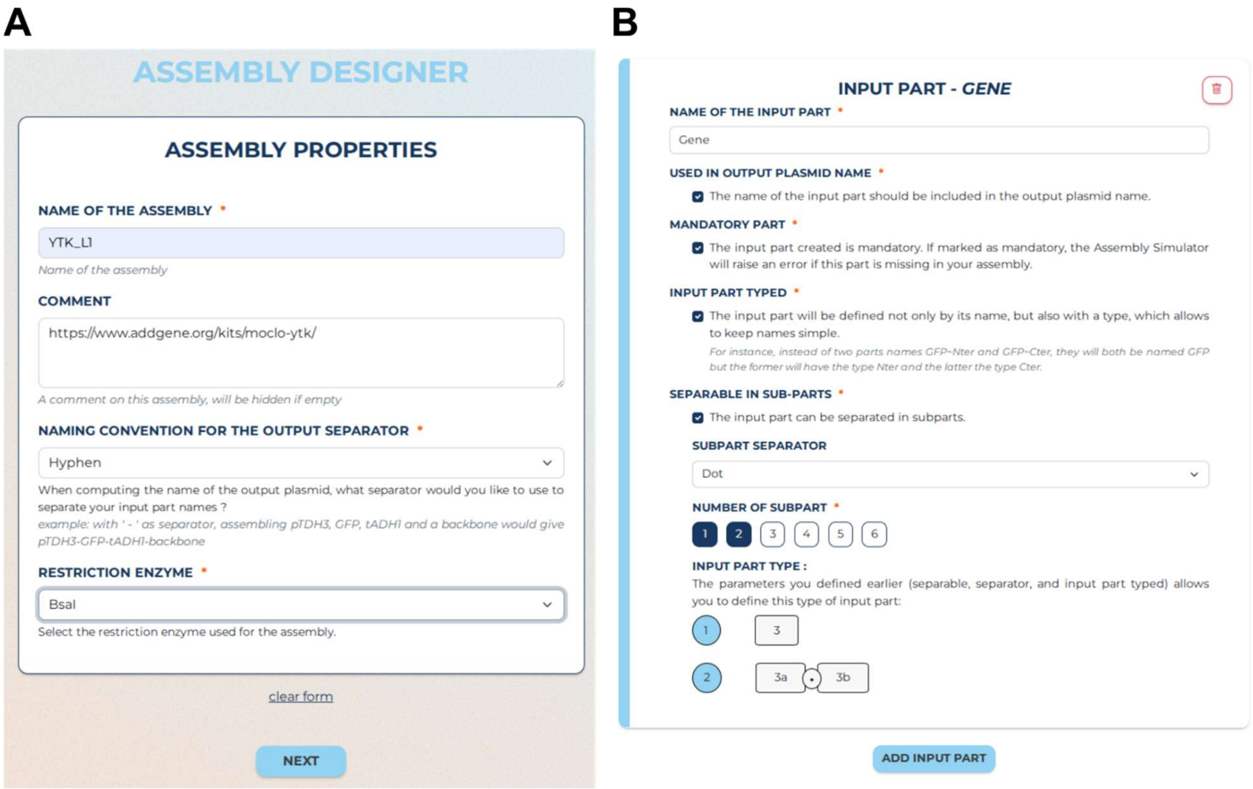

Figure 10: Interface of the Assembly Designer module*. (A) Definition of the assembly properties. (B) Definition of the input part properties with the "Gene" example. Users can add as many input part as needed, and for each define their characteristics.*

- Types of parts and subparts cannot be edited in the web application. They are by default 1, 2 … for part and 1a, 1b… for subparts. Users need to modify them in the *template.xlsx* file once downloaded. Only numbers and letters are accepted.

2. Prepare lab data
   - *IP_mapping.csv* which maps genetic part names with Genbank file names (can be omitted if Genbank file names are used when specifying genetic designs).
   - *SequenceDB.zip* archive with the Genbank files (only .gb files).

Campaign

1. Fill in *template.xlsx* with genetic designs and save it as a campaign file
   - The type of each output plasmid is set manually in the "OutputType" column. For most MoClo system, it depends on the backbone used. Alternatively, it can depend on the connectors as in the YTK[1].
2. Upload on Assembly Simulator the filled template, then the lab data
3. Run the simulation to generate campaign data
   - Plasmid maps and input file of Slowpoke are generated by default
   - To compute dilutions, input plasmid concentrations are needed. They can be added in *IP_mapping.csv* from the beginning, or added in *input-plasmid-concentrations.csv* downloaded from the "Compute dilution" section of the result page
   - Upload the primer database if colony PCR is simulated

Additional information and example of datasets are available on https://insillyclo.pasteur.cloud/tutorial.

Text S6: InSillyClo with other MoClo systems

Public assemblies for several MoClo kits are available on the web application (https://insillyclo.pasteur.cloud/assembly/). Additionally, we generated the input files (*template.xlsx, iP_mapping.csv* and *SequenceDB.zip*) and a demo of campaign for some of them. When downloading *template.xlsx* from the web application, part types are numbers (1, 2 …) so users must change them if needed. We present below possible typing systems for each of these MoClo kits. They are all hierarchical so input and output types of each level are made compatible. The associated files are available at https://gitlab.pasteur.fr/insillyclo/insillyclo-web/-/tree/main/src/InSillyCloWeb/test_data/moclo_assembly_template?ref_type=heads.

MoClo Plant Part Kit[2]

This system allows typical Transcriptional Unit (TU) assembly in the level 1, followed by multi-Transcriptional Unit (mTU) assembly in level 2. Modularity of the TU structure is well captured by using subparts. For instance, the promoter region can be made one part (Pro5U), two subparts (Pro and 5U), or three subparts (Pro, 5Uf, NT1). This system allows the assembly of multiTU together through an iterative process with M and P levels. We think that “untyped” (ie raw) assemblies are more suited for these levels since designs become highly custom with the possibility to have any TU at the start or at the end of an assembly. In addition, standardized names of these multiTU plasmids can be rather long, making more convenient to use directly Genbank file names in design specification, as shown in Text S2. Of note, the first M assembly is similar to a Level 2 assembly.

Assembly level	Input part types	Output part types
Level 1	-Pro5U, [Pro, 5U], [Pro, 5Uf, NT1]<br>-CDS1, [SP, CDS2]<br>-CT<br>-3UTer, [3U, Ter]<br>-BL1 (Backbone L1)	TU1, TU2, TU3, TU4, TU5, TU6, TU7
Level 2/M1	-TU1<br>-TU2<br>-TU3<br>-TU4<br>-TU5<br>-TU6<br>-TU7<br>-ELL2/ELLM (End-Linker L2/M)<br>-BL2/BLM (Backbone L2/M)	mTU

Table 1: Typing system for the Plant Part Kit Moclo.

EcoFlex Moclo[3]

EcoFlex has the typical Level 1 and Level 2 assemblies for construction of TU and mTU. As the Plant Part Toolkit, it allows to assemble together several mTUs, with the difference of being a unique additional assembly (i.e. Level 3 instead of Level M/P) with a simpler typing system.

Assembly level	Input part types	Output part types
Level 1	-1, [1a, 1b], [1a, 1b1, 1b2] (promoter, RBS and signal peptide)<br>-2 (CDS)<br>-3 (Terminator)<br>-BL1 (Backbone L1)	TUA, TUB, TUC, TUD, TUE
Level 2	-TUA<br>-TUB<br>-TUC<br>-TUD<br>-TUE<br>-BL2 (Backbone L2)	mTUA, mTUB, mTUC, mTUD
Level 3	-mTUA<br>-mTUB<br>-mTUC<br>-mTUD<br>-BL3 (Backbone L3)	mTU

Table 2: Typing system for the EcoFlex Moclo.

Cidar Moclo[4]

This Moclo kit is similar than the EcoFlex in that it allows 3 level of assemblies but the level 0 design is different with promoters and terminator containing TU-specific overhang. Another difference is the reuse of the level 1 backbone in the level 3.

Assembly level	Input part types	Output part types
Level 1	-1 (promoter)<br>-2 (RBS)<br>-3 (CDS)<br>-4 (Terminator)<br>-BK (Backbone Kanamycin)	TUKae, TUKef, TUKfg, TUKgh
Level 2	-TUKae<br>-TUKef<br>-TUKfg<br>-TUKgh<br>-BA (Backbone Ampicillin)	TUAae, TUAef, TUAfg, TUAgh

Level 3	-TUAae<br>-TUAef<br>-TUAfg<br>-TUAgh<br>-BK (Backbone Kanamycin)	mTU

Table 3: Typing system for the Cidar Moclo.

Vnat

The Vnat collection allows the assembly of TUs and mTUs. A particular feature is the possibility to create operons by using special connectors bypassing the presence of promoter and terminator between two consecutive genes. The use of connectors in the level 2 assembly leaves the door open to continue with level 3 construct.

Assembly level	Input part types	Output part types
Level 1	-5conOp, [5con, Prom]<br>-RBS<br>-CDS, [Nter, CDS2]<br>-3conOp, [Term, 3con], [Cter, Term2, 3con]<br>-Ori<br>-Bb1	TU1, TU2, TU3, TU4, TU5
Level 2	-5con<br>-TU1<br>-TU2<br>-TU3<br>-TU4<br>-TU5<br>-Ori2<br>-Bb2	mTU

Table 4: Typing system for the Vnat collection.

Text S7: Local implementation with the command line tool

We describe below the workflow taking as example the yeast display campaign. The full documentation is available at https://insillyclo.pages.pasteur.fr/insillyclo-cli/ and test datasets can be found at https://gitlab.pasteur.fr/insillyclo/insillyclo-web/-/tree/main/src/InSillyCloWeb/test_data/tutorial/Cli_assembly?ref_type=heads.

Setup

1. Get a local implementation of python
   - Download miniforge at https://conda-forge.org/miniforge/, and keep the "create shortcut" option during the installation.
2. Open miniforge prompt (from the start menu in Windows)
3. Run `pip install insillyclo`

Template command for the initialization

1. Run the *template* command

```
insillyclo template C:\Users\Henri\Documents\InSillyClo\Template_YTK_L1_BASE.xlsx
--name "YTK_L1"
--enzyme "BsaI"
--separator -
--input-part ConL
--input-part Promoter
--input-part Gene
--input-part Terminator
--input-part ConR
--input-part Backbone
```

Note: it needs to be given as a single line

2. If needed, edit the template file in a spreadsheet program to adapt input part parameters

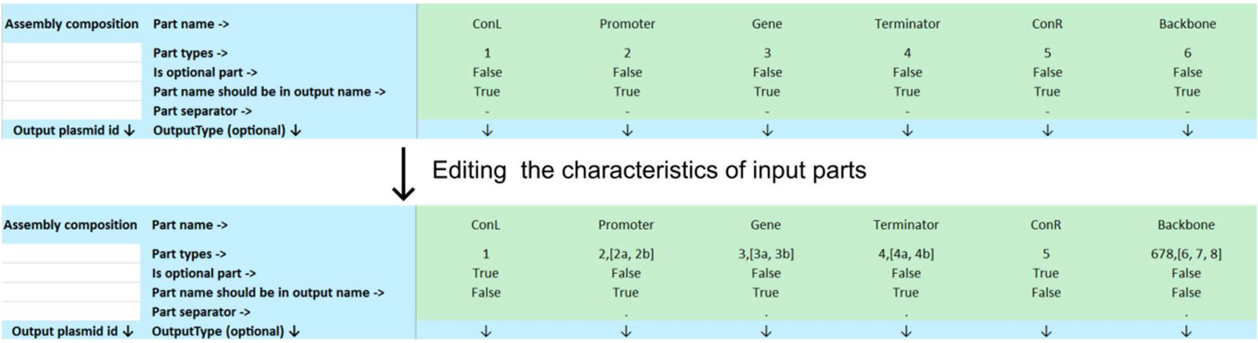

Figure 11: Template editing with a spreadsheet program.

- Part types are modified to add sub-parts and to correspond the grammar of the Moclo system.
- ConL and ConR are made optional.
- ConL, ConR and Backbone are removed from the output name.
- Part separator is set to be the dot "." or none.

3. Prepare lab data (*iP_mapping.csv*, plasmid repository, and table of primers)

As opposed to *SequenceDB.zip* in the web application, the plasmid repository is the folder containing the Genbank files (only .gb). It does not need to be zipped.

***Simulate* command for the campaign**

1. Fill in the template with genetic designs

2. Run the *simulate* command

```
insillyclo simulate
--input-template-filled C:\Users\Henri\Documents\InSillyClo\Campaign_YTK_display_cli.xlsx
--input-parts-file C:\Users\Henri\Documents\InSillyClo\iP_mapping_YTK.csv
--plasmid-repository C:\Users\Henri\Documents\InSillyClo\SequenceDB_YTK
--output-dir C:\Users\Henri\Documents\InSillyClo\CampaignData_display_cli
--restriction-enzyme-gel NotI
--primer-pair P29,P30
--primers-file C:\Users\Henri\Documents\InSillyClo\DB_primer.csv
--default-mass-concentration 200
```

Users of the command line tool need to specify all the results they want directly in the *simulate* command. It is possible to simulate PCR with several primer pairs, and digestion with several restriction enzymes (see details at https://insillyclo.pages.pasteur.fr/insillyclo-cli/simulate.html#insillyclo-simulate).

For users reluctant to upload their files on the server in the Assembly Simulator module, it is still possible to combine the web application and the command line tool. Concretely, the Assembly Designer module is used to generate the *template.xlsx* file, followed by the *simulate* command of the command line tool which handles files locally.

References

(1) Lee, M. E.; DeLoache, W. C.; Cervantes, B.; Dueber, J. E. A Highly Characterized Yeast Toolkit for Modular, Multipart Assembly. *ACS Synth. Biol.* **2015**, *4* (9), 975–986. https://doi.org/10.1021/sb500366v.

(2) Engler, C.; Youles, M.; Gruetzner, R.; Ehnert, T.-M.; Werner, S.; Jones, J. D. G.; Patron, N. J.; Marillonnet, S. A Golden Gate Modular Cloning Toolbox for Plants. *ACS Synth. Biol.* **2014**, *3* (11), 839–843. https://doi.org/10.1021/sb4001504.

(3) Moore, S. J.; Lai, H.-E.; Kelwick, R. J. R.; Chee, S. M.; Bell, D. J.; Polizzi, K. M.; Freemont, P. S. EcoFlex: A Multifunctional MoClo Kit for E. Coli Synthetic Biology. *ACS Synth. Biol.* **2016**, *5* (10), 1059–1069. https://doi.org/10.1021/acssynbio.6b00031.

(4) Iverson, S. V.; Haddock, T. L.; Beal, J.; Densmore, D. M. CIDAR MoClo: Improved MoClo Assembly Standard and New E. Coli Part Library Enable Rapid Combinatorial Design for Synthetic and Traditional Biology. *ACS Synth. Biol.* **2016**, *5* (1), 99–103. https://doi.org/10.1021/acssynbio.5b00124.
